# Electrochemically driven mechanical energy harvesting

**DOI:** 10.1038/ncomms10146

**Published:** 2016-01-06

**Authors:** Sangtae Kim, Soon Ju Choi, Kejie Zhao, Hui Yang, Giorgia Gobbi, Sulin Zhang, Ju Li

**Affiliations:** 1 Department of Materials Science and Engineering, Massachusetts Institute of Technology, Cambridge, Massachusetts 02139, USA; 2 Department of Mechanical Engineering, Massachusetts Institute of Technology, Cambridge, Massachusetts 02139, USA; 3 Department of Nuclear Science and Engineering, Massachusetts Institute of Technology, Cambridge, Massachusetts 02139, USA; 4 Department of Engineering Science and Mechanics, Pennsylvania State University, University Park, Pennsylvania 16802, USA; 5 Politecnico di Milano, Department of Mechanical Engineering, Milan, 20156, Italy

## Abstract

Efficient mechanical energy harvesters enable various wearable devices and auxiliary energy supply. Here we report a novel class of mechanical energy harvesters via stress–voltage coupling in electrochemically alloyed electrodes. The device consists of two identical Li-alloyed Si as electrodes, separated by electrolyte-soaked polymer membranes. Bending-induced asymmetric stresses generate chemical potential difference, driving lithium ion flux from the compressed to the tensed electrode to generate electrical current. Removing the bending reverses ion flux and electrical current. Our thermodynamic analysis reveals that the ideal energy-harvesting efficiency of this device is dictated by the Poisson's ratio of the electrodes. For the thin-film-based energy harvester used in this study, the device has achieved a generating capacity of 15%. The device demonstrates a practical use of stress-composition–voltage coupling in electrochemically active alloys to harvest low-grade mechanical energies from various low-frequency motions, such as everyday human activities.

## Abstract (web summary)

There is intensive research underway into the development of various mechanical energy harvesters. Here, the authors report an electrochemically driven mechanical energy harvester that uses the stress-induced potential difference of lithiated silicon electrodes to generate continuous electricity.

## Graphical abstract

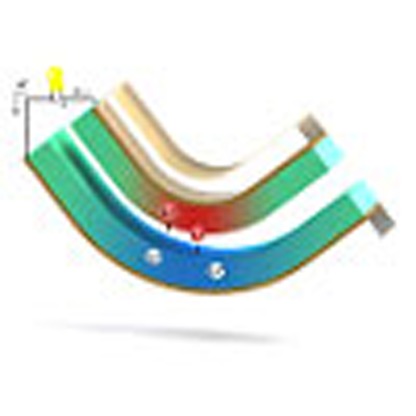

Efficient energy-harvesting devices, which convert energies otherwise wasted to electricity, help decentralize power generation and reduce the distance of electricity transmission. During the last decade, enormous efforts have been dedicated to the development of a variety of energy harvesters, capable of harvesting energy of various forms^1^,^2^,^3^,^4^,^5^. In mechanical energy harvesting alone, several types of energy generators have been demonstrated, such as piezoelectric^6^, electrokinetic^7^,^8^,^9^ or triboelectric generators^10^, and enabled a wide range of applications^11^,^12^,^13^,^14^,^15^,^16^. Advances in processing techniques such as virus-directed designs^17^ or block copolymer self-assembly^18^ have also been reported. However, these energy generators are most efficient for vibrational energy harvesting at a relatively high frequency (∼20–100 Hz), and inherently limited in the low-frequency regime (0.5–5 Hz) where everyday human activities such as walking take place.

Herein we report a new type of mechanical energy harvester operative in the low-frequency regime. The device uses the stress-composition coupling in electrochemically active materials, such as partially Li-alloyed Si or Ge (refs 19, 20, 21). The coupling between mechanical stress and lithiation thermodynamics and kinetics^22^ has been widely recognized in high-capacity anodes of lithium ion (Li^+^) batteries, but was usually regarded as an adverse effect^19^,^23^,^24^. Here we demonstrate that mechanical bending induces different stress states in two identical partially Li-alloyed Si electrodes, which drives Li^+^ migration and generates electricity. The prototype generator demonstrates power density of 0.48 μW cm^−2^ at 0.3 Hz. Our thermodynamic and mechanics analyses lay a theoretical foundation for the device design and optimization.

## Results

### Working principle and device design

**Figure 1:**
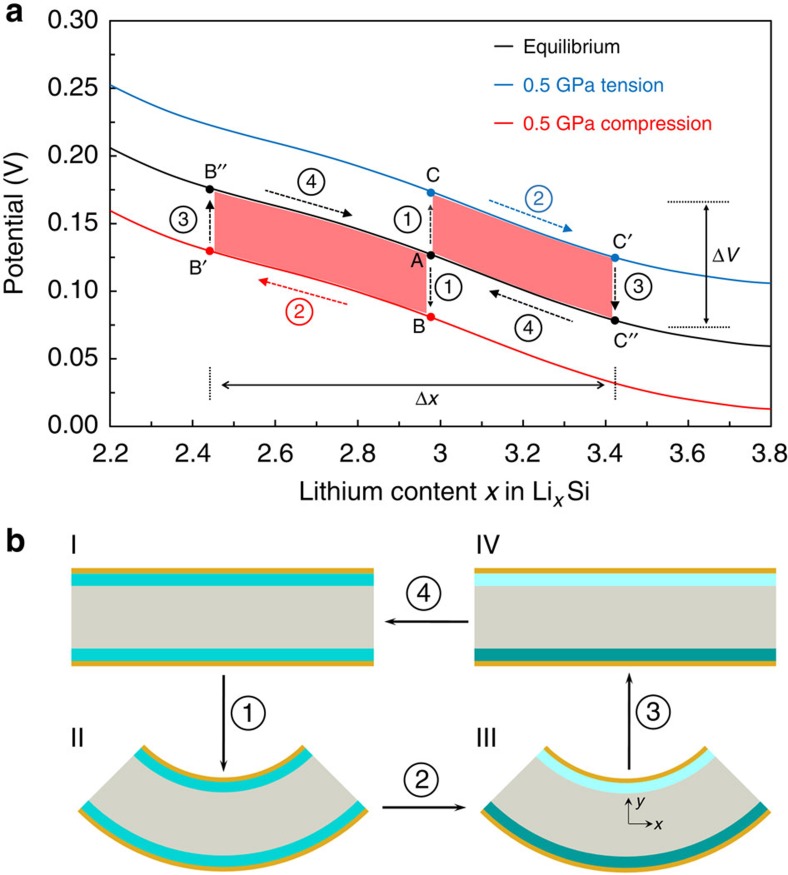
The working principle behind the mechanical energy harvesting device. (**a**) Thermodynamic perspective on bending-unbending cycle. On introducing different stress states by bending, a chemical potential difference develops between two electrodes. When the electrodes are connected by an external circuit, new equilibrium under the stress states are established by Li^+^ migration. Once the stresses are removed, the lithiation states return back to the original equilibrium state. The area covered by this cycle in red measures the energy output obtained. (**b**) Schematics of the cross-section of the device in operation.

Figure 1 illustrates the working principle of the energy harvester, consisting of two partially Li-alloyed electrodes sandwiching an electrolyte. In the initial stress-free condition, the two electrodes are isopotential (point A in Fig. 1a and I in Fig. 1b). Bending the device generates net tension in one electrode and compression in the other (points B and C in Fig. 1a and II in Fig. 1b). The asymmetric stress creates a chemical potential difference that drives Li^+^ ion migration from the compressed to the tensed electrode through the electrolyte (see Supplementary Note 1 for the analysis). At the same time, to maintain charge neutrality, electrons flow in the outer circuit, also from the compressive to the tensile sides, generating electrical power. The Li^+^ migration continues until the potential difference vanishes (points B′ and C′ in Fig. 1a and III in Fig. 1b), establishing new equilibrium states on the two electrodes with different Li concentration. When the external stresses are removed by unbending the device, the chemical potential shifts on the electrodes (from point B′ to point B′′ and from point C′ to point C′′ in Fig. 1a and III–IV in Fig. 1b). The difference in lithium concentration between the electrodes drives Li^+^ ion migration in the opposite direction (from point B′′ and C′′ to A in Fig. 1a and IV–I in Fig. 1b), thus discharging the device. The device goes back to its original equilibrium state and may go through this cycle multiple times provided that it operates in the viscoelastic regime without any irreversible damage. The electrical energy generated is equivalent to the potential difference multiplied by the amount of Li^+^ migrated, as illustrated as red-colored area in Fig. 1a.

**Figure 2:**
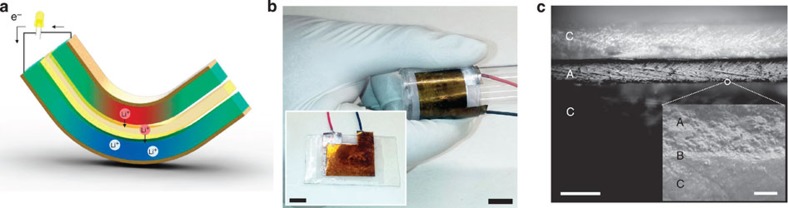
A prototype of the mechanical energy harvester. (**a**) Schematic view of the device design. Compressed region is illustrated in red while the tensile region is illustrated in blue. Lithium ions migrating from the compressed plate to the tensile plate are shown with arrows. The electrolyte soaked separator is drawn in yellow. (**b**) An image of the actual device with a bending unit. Both scale bars indicate 1 cm. (**c**) Cross sectional image of the device showing polypropylene electrolyte layer (A in the figure), Li_*x*_Si electrode on Ag current collector (B in the figure) and polyimide adhesion layer (C in the figure). The scale bars on the left and right indicate 40 and 2 μm, respectively.

Guided by this vision, we developed a prototype generator, consisting of two identical electrodes sandwiching a separator soaked with electrolyte. We used amorphous Li_*x*_Si (*x*∼3.1) thin film as the electrodes for its mechanical flexibility and reasonable lithiation and delithiation rates^25^. Ethylene carbonate mixed with ethyl methyl carbonate, LiPF_6_ and micro-porous polypropylene monolayer^26^ were used as the electrolyte, lithium salt and separator, respectively. We selected polyimide as the substrate to which the electrodes attach and Ag current collectors for their strong adhesion and stretchability^27^. The polyimide substrates were encapsulated by castable rubber, such as polydimethylsilane (PDMS) or polyurethane. Figure 2 shows the schematics of the device and the atomistic view of the active region. Each Li_*x*_Si electrode is 249 nm thick, about two orders of magnitude thinner than the separator layer (25 μm). The thin-film configuration of the device allows large-curvature bending. On bending, the top, compressed electrode becomes an anode, while the bottom, tensed electrode a cathode. The device functions as an energy harvester, and because stress-driven Li^+^ ion diffusion conforms to the Onsager linear-response behaviour, it is expected to exhibit a decent efficiency even with miniscule loads (that is, no threshold behaviour), converting mechanical energy input into electrical energy output.

### Mechanics analysis of the device

Mechanics analysis provides insight into the energy conversion efficiency. Figure 1b illustrates the bending geometry. Bending the thin-film device generates compressive and tensile strains of equal magnitude 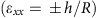 on the top and bottom electrodes, respectively, where *R* is the radius of curvature and *h* the half thickness of the thin-film device. The stress state is obtained under the assumption of elastic deformation. Assuming a plane-stress condition along the *y* direction (see Fig. 1b for the coordinates), the stress on the bottom electrode can be written as follows,


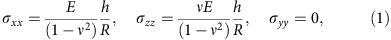


where *E* is the Young's modulus and *ν* the Poisson's ratio. The chemical potential difference between the two electrodes is only related to the difference in the hydrostatic stress 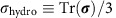



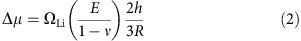


Where Ω_Li_=14.95 Å^3^ is the estimated partial molar volume of Li in Li_*x*_Si (refs 28, 29). This gives a pressure sensitivity of 93 mV per GPa, close to a recent experimental measurement of 110 mV per GPa (ref. 24).

It is noted that the deviatoric part of the stress tensor 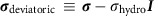 does not couple to the chemical potential, where ***I*** is the identity tensor. Instead, the deviatoric stress ***σ***_deviatoric_ induces shear deformation which is volume conservative, thus does not generate electrical energy. The total strain-energy (*U*_strain_) can be decoupled into the hydrostatic part and the deviatoric part, as:


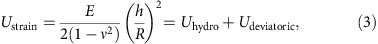


with


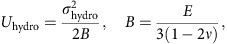


where *B* is the bulk modulus. Only the hydrostatic component *U*_hydro_ can be used for electricity generation, which also varies on lithium insertion and extraction. Assuming all of *U*_hydro_ can be used for electricity generation, while all of *U*_deviatoric_ is wasted, an ideal efficiency can be expressed as


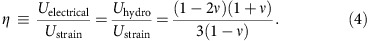


For *v*=0.25 (ref. 30), an idealized efficiency of 27.8% is obtained. Interestingly, the energy conversion efficiency is independent of the Young's modulus, and maximizes at *v*=0. The total amount of lithium that is expected to transport across the electrolyte to completely relax ***σ***_hydro_ is


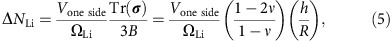


where *V*_one side_ is the volume of one Li_*x*_Si electrode affected by the radius of curvature and 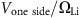 the amount of Li present in the affected volume determined by previous reports^28^. It is noted that a thicker electrode increases the amount of migrating lithium and hence the capacity, however, it may also increase the probability for developing structural inhomogeneities such as cracks during lithiation or bending.

### Electrical energy output of the device

**Figure 3:**
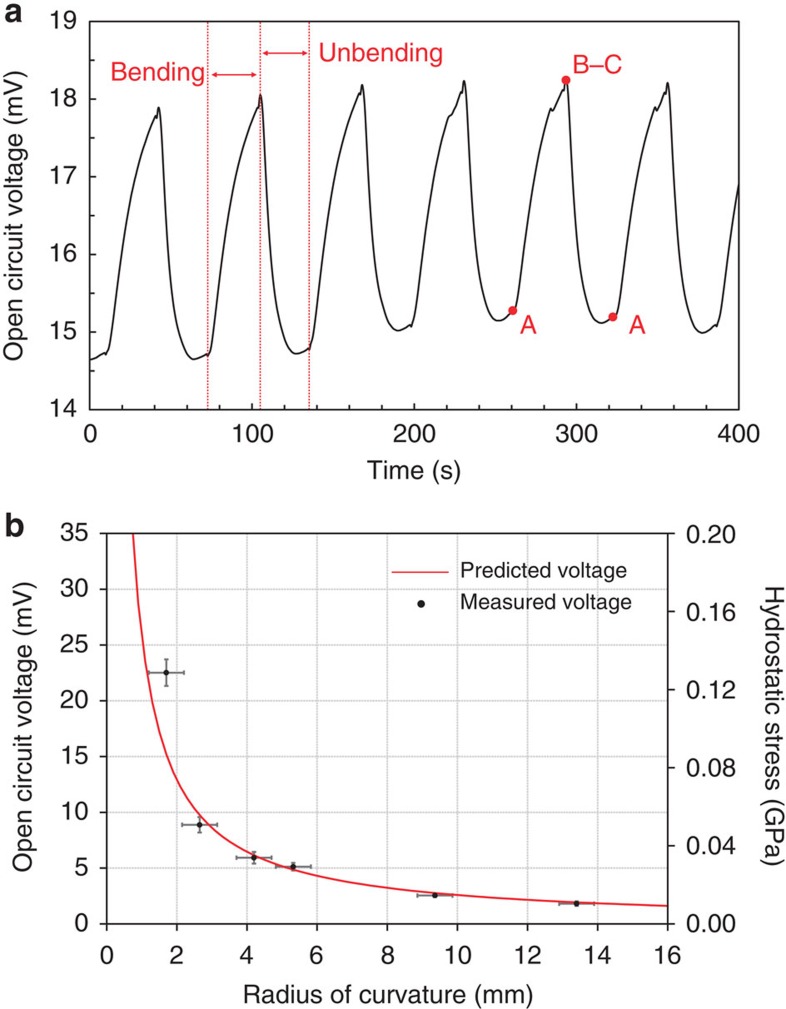
Open circuit voltages measured during bending tests. (**a**) The open-circuit voltage measured from simple bending of the device. The measured values show clear voltage peaks during bending and releasing the device, each with 30 s interval. Each alphabetical points correspond to the bending geometry illustrated in Fig. 1b. (**b**) The predicted open-circuit voltage and hydrostatic stress according to the radii of curvature, operated in the elastic regime. s.e. resulted from at least five measurements for each radius of curvature is included. The measured voltage values agree well with the predicted values.

Just like photovoltaic energy harvesters, one could characterize a mechanical energy harvester by open-circuit voltage or short-circuit current. Figure 3a shows the open-circuit voltage, obtained by bending the device in the same direction for 30 s and resting the device for another 30 s with bending force released. The applied radius of curvature was ∼1 cm, corresponding to a maximum tensile stress of 0.018 GPa generated in the electrodes. Since the yield stress of amorphous Li_*x*_Si is ∼1 GPa, the material deforms within its elastic regime^31^. We note that there exists a background potential (nonzero rest potential), which might be due to the side reactions such as a solid electrolyte interface layer formation or any unintentional inhomogeneity in composition between the two electrodes. The open-circuit voltage increases on bending and recovers its resting potential once the bending force is released. This trend is consistent with the relationship between the chemical potential difference and the applied radius of curvature (*Δμ*∼*R*^−1^), shown in equation (2). We used experimentally measured value of Young's modulus (25 GPa) for lithiated amorphous silicon thin film, interpolated with rule of mixture for the specific composition we used^32^. Table 1 shows the predicted hydrostatic stress, voltage and measured voltage values at six different radii of curvature. The measured open-circuit voltages agree well with the predicted values.

**Figure 4:**
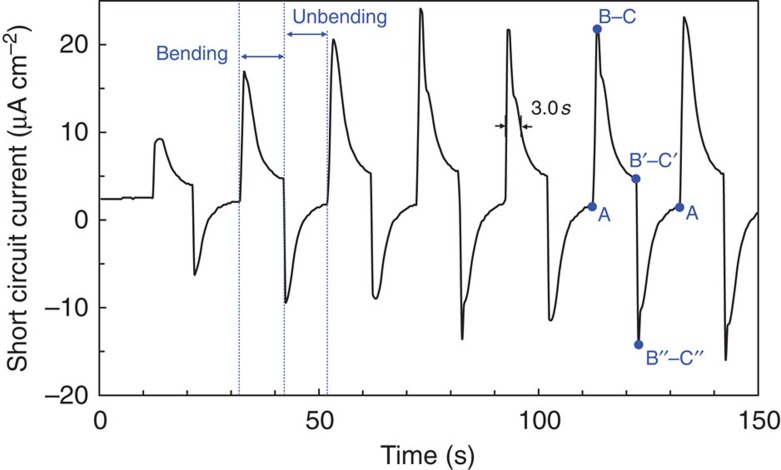
Short circuit current density during bending tests. Bending was maintained at 2.0 mm radius of curvature with 10 s intervals. The positive peaks correspond to the current during bending and negative peaks to the current during unbending.

Figure 4 shows the measured short-circuit current as the device is bent with a radius of curvature of 0.2 cm and then relaxed by releasing the bending forces. The bending and relaxation periods were 10 s each. Bending induces a sharp rise in the current, suggesting the stress-driven Li^+^ migration inside. When holding the bending at a fixed radius of curvature, the current signal quickly reaches a maximum beyond which it decays gradually. This decay is due to the cancellation between the externally applied bending stress and Li insertion/extraction-induced stress in the electrodes. Specifically, externally applied bending creates tensile stress on one electrode and compressive on the other, generating a chemical potential difference that drives Li diffusion from the compressive to the tensile sides against the Li concentration gradient. At the same time, Li extraction from the compressed electrode and insertion into the tensed electrode attenuate both the compressive and tensile stresses, corresponding to a reduced driving force for Li^+^ diffusion and to the gradual current decay. The current signal vanishes when the chemical potential between the two electrodes vanishes. The full width at half maximum (time for peak current value to drop to half) was 3.0 s on average, which is two orders of magnitude greater than a typical piezoelectric device in similar geometry, promising the applicability of the device in the low-frequency regime^33^.

On releasing the device from external bending force, Li insertion/extraction-generated stress difference along with the Li concentration gradient in the electrodes serves as the driving force for the backward Li^+^ migration, corresponding to a sharp current increase in the opposite direction. As Li^+^ migration continues, the stress and concentration difference between the two electrodes drop, so as the driving force for Li^+^ diffusion, leading to a reduced current signal. The current signal vanishes when the two electrodes recover to their original, isopotential state. We also performed measurements by alternating the bending directions and the results were consistent with the trends described above, as illustrated in Supplementary Note 2.

The amount of Li^+^ ions that migrate during each bending cycle is equivalent to the area under a current peak. In Fig. 4, the area under a peak is ∼73 μC. The volume of the affected area during the experiment is estimated to be 1.56 × 10^−5^ cm^3^. (The bending geometry during experiment is shown in Supplementary Note 3.) According to the analysis above, the expected Li^+^ ion migration is equivalent to 487 μC. The reversible lithium migration of 73 μC is ∼15% of the theoretically predicted amount. The reduced generating capacity per cycle may be caused by ‘self-discharging', that is, the electrons diffuse out of the bent region to the nearby flat regions within the same Li_*x*_Si electrode without going to the external circuit, which actually causes the flat regions to curve. Such self-discharging behaviour is predicted to occur whenever there are unequal bending curvatures in the lateral direction (gradient in the bending curvature) and is expected to be a significant cause in low experimentally measured efficiency of the device. (The experimentally measured efficiency is estimated in Supplementary Note 4.) This internal electrochemical dissipation by self-discharging can be greatly reduced by applying a uniform radius of curvature throughout the device.

### Device durability during repeated bending test

**Figure 5:**
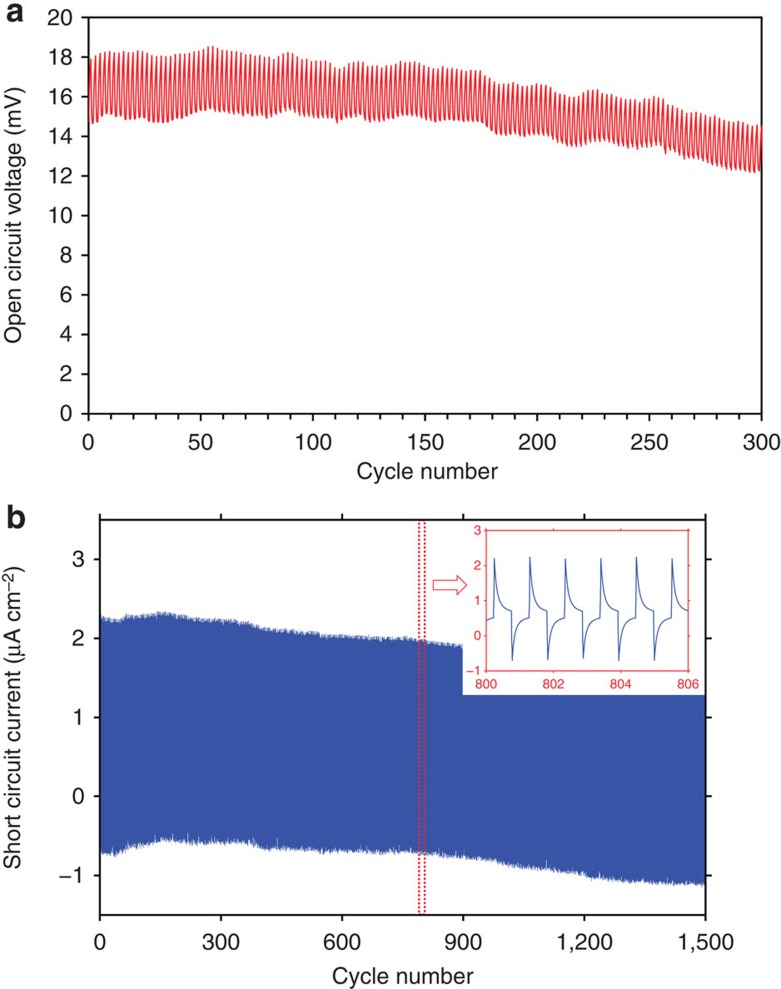
Electricity generated during repeated bending tests. (**a**) The open-circuit voltage at 10 mm radius of curvature and (**b**) short-circuit current collected during repeated bending tests at 4.0 mm radius of curvature. The nested figure shows the zoomed-in view of the 800th–807th bending cycle.

Figure 5 shows the data from two kinds of repetitive bending fatigue tests. In repeated open-circuit voltage tests, we observe that not only the peak height but also the background voltage are reduced over cycling, despite bent at moderate radius of curvature (10 mm). This is due to the damage accumulation in the electrodes. In open-circuit voltage measurements, lithium ions cannot migrate between the electrodes to relax the stresses developed in the electrodes. As a result, damages are accumulating in the electrodes in the form of pore formation, or even fracture. Since the films can no longer sustain the elastic stress, the peak heights decrease. In addition, it is expected that the electrode under repetitive tension exhibits different damage accumulation from that under repetitive compression. This difference results in the potential difference between the two electrodes and changes the background potential. In repeated short-circuit current tests, in contrast, we observe no major degradation in the peak height. The data in Fig. 5b shows reliable current generation during 1,500 repeated bending cycles. The background current is slowly moving towards zero during cycling in a stable manner. Data from repeated bending under different radii of curvature is available in Supplementary Note 5. These results indicate that the electrode materials maintain their homogeneity during cyclic loading of 1,500 cycles. The reliable performance of the device during short-circuit fatigue test is enabled as the migrating lithium ions act as effective stress reliever in the electrode, much the same way as in Nabarro–Herring creep^34^. It might thus be possible to adapt our active mechanical–passive electrochemical fatigue tests as a diagnostic tool to characterize damage creation, repair and accumulation inside electrode materials, as a complementary technique to the electrochemically driven battery cycling testing paradigm that monitors voltage-induced mechanical strain (such as film curvature change).

Since our generator should operate in the elastic regime to minimize damage accumulation, the yield surface of electrode material sets the limit on the voltage output. The atomic volume of lithium is known to be approximately constant in a wide range of lithium alloys independent of lithium content^28^, and thus, an alloy with the greatest yield strength promises the highest voltage output. It is also noteworthy that our device does not cause composition change nearly as large as that in battery electrode reactions, therefore many candidate alloys that do not cycle well as battery electrodes may still be excellent candidates for our generator device. Alloys of larger ions such as sodium or potassium may also be used to provide higher voltage output, as a higher atomic volume promises higher voltage.

## Discussions

Since the electricity generation is driven by Li^+^ ion diffusion, the effect of bending rate and frequency can be understood based on the characteristic time scales of Li^+^ ion diffusion. While there are time scales for mechanical equilibrium and diffusion equilibrium, the mechanical equilibrium in the elastic range is established very fast. Thus, potential bending rate effect comes from the competition between applied strain rate and diffusion rate. Using a typical value of *D*_Li_=10^−10^ cm^2^ s^−1^ and letting 250 nm be the characteristic diffusion distance, we estimate that the characteristic diffusion time is about 6 s. As long as strain rate exceeds Li^+^ ion diffusion rate characterized by the width of the short-circuit current signal, we observe identical current curves with similar full width at half maximum. If strain rate is slower, a current signal with extended width and reduced peak current is expected. In either case, the total energy output or repeatability is not expected to change owing to the bending rate. Similarly, slow bending frequency would allow sufficient time for Li^+^ ions to migrate between the electrodes and does not affect the output energy. These are demonstrated experimentally and are illustrated in Supplementary Note 6. If bent at a frequency so high that Li^+^ ions do not have sufficient time to migrate between electrodes, the electrodes are not able to relieve stress by lithium insertion/extraction and will eventually fail by fatigue. This is in part equivalent to low temperature condition in Nabarro–Herring creep. This frequency, however, far exceeds the human activity timescale. The device is therefore not well suited for vibrational energy harvesting at high frequency (≫100 Hz).

The energy output generated from the device in general exhibits a greater amount of current and less voltage when compared with the ceramic piezoelectric generators. Table 2 shows the comparison of peak power and energy output operated at 0.3 Hz frequency for known piezoelectric generators and our device. The piezoelectric generators cited in Table 2 have similar geometry to our device and the difference in energy output comes mainly from the material properties. The comparison is based on the area of the thin-film generators without considering film thickness, as the microstructure of the materials as well as the effect of thickness on performance differ significantly among the reported generators. In Table 2, PMN-PT (ref. 11) refers to (1−*x*)Pb(Mg_1/3_Nb_2/3_)O_3_–*x*PbTiO_3_ and is a single crystalline thin film, while BaTiO_3_ (ref. 33) is a nanoparticle composite. KNLN (ref. 35) refers to 072(K_0.480_Na_0.535_)NbO_3_–0.28LiNbO_3_ and is a nanoparticle composite. Peak power is considered to be the direct product of short-circuit current and open-circuit voltage. The energy output at 0.3 Hz is calculated, assuming that the average peak width (full width at half maximum) of piezoelectric generators is 100 ms and that the peak width of our device is 3 s. As shown in Table 2, the peak power for our device is in general less than those of piezoelectric generators. Nevertheless, when operated under low frequency such as 0.3 Hz, the amount of energy generated per second is comparable to the best non-lead containing piezoelectric generators in the same form factor; our device outperforms those made of BaTiO_3_ or KNLN by at least one order of magnitude. The PMN-PT-based generators and ZnO (ref. 15) nanowire generators outperform our device; nevertheless, these devices are elaborately optimized. PMN-PT generators consist of 20-μm-thick single crystalline thin film, grown and processed from an ingot of the material and sapphire substrate. The ZnO nanowire generators were optimized by coating the surface with layer-by-layer self-assembled polymer layer. Considering that both generators have gone through a significant amount of optimization, our device, with further optimization in material selection and architecture design, holds promise as an efficient energy harvester at low frequencies.

We have developed a novel type of mechanical energy harvester based on the fact that lithium ions have finite size (14.95 Å^3^), and thus would migrate across an electrolyte membrane under a pressure difference–akin to reverse osmosis in seawater desalination. But because lithium ions carry +*e* charge, it would drive electron flow in the outer circuit, same as how a typical battery would work. The device achieves long current pulse duration, which has not been achieved by other types of mechanical energy-harvesting devices. Owing to this characteristic, the device exhibits higher average energy output than most other piezoelectric generators when operating at low frequencies. This work also opens avenues for optimizing electrochemical devices coupled to mechanical stress for sensing and actuation, as well as the development of active mechanical–passive electrochemical tests as an alternative diagnostic protocol to study damage creation and accumulation in electrochemomechanically active solids.

## Methods

### Flexible substrate choice and electrode deposition

We chose Kapton Polyimide (PI) film as the flexible substrate. PI films provide flexibility, chemical compatibility with lithium ion electrolytes and strong adhesion between the film and electrode materials^27^. The device fabricated on several other flexible substrates resulted in electrode film cracking, little electrical conductivity or delamination during electrochemical lithiation step. On PI substrate, current collector consisting of 15 nm Cr and 100 nm Ag is deposited using electron beam evaporator at 1 and 2 Å s^−1^ rate, respectively. On Ag thin film, amorphous Si electrode of 75 nm (Kurt Lesker, p-type) is deposited using e-beam evaporator. Thick silicon film (75 nm) was selected carefully to avoid potential structural inhomogeneities such as pores and cracks according to previous studies^25^. A 3 nm Ti adhesion layer was used between Ag and Si. A small region of Ag was left free of silicon for electrical connection.

### Electrochemical lithiation

Lithium was inserted into silicon by electrochemical lithiation. An electrochemical cell consisting of the Si on PI, electrolyte (ethylene carbonate:ethyl methyl carbonate=1:1 with LiPF_6_, Novolyte) soaked separator (Celgard 2500) and thick Li foil (Alfa Aesar, 1.5 mm thick) was lithiated using Gamry Reference 3000 potentiostat at 0.5 C, up to 80% of the theoretical capacity (2,880 mAh g^−1^ out of 3,600 mAh g^−1^). This corresponds to the film composition of approximately Li_3.1_Si. The low cutoff voltage was set to 0.05 V to avoid nucleation of crystalline phase^36^ and ensure that the film remains as homogeneous as possible as an amorphous Li–Si alloy. Previous studies have reported that amorphous Si thin films lithiated at C/10 remain smooth to nanometre scale^37^. Control experiments performed with films lithiated at C/2 and C/10 produced identical result within the scope of error and all lithiation was performed at C/2 in this study.

### Device assembly and testing

The electrode prepared above was mounted onto thin PDMS film (Sylgard 184, Dow Corning Chemical) for easy handling and encapsulation. PDMS films were prepared by mixing the elastomer with hardener at 10:1 mass ratio and curing at 65 °C for 2 h. Paraffin wax was dissolved onto PDMS as illustrated in previous report^38^ to avoid gas and vapour permeability in PDMS. When extra inhibition to gas permeation was required, for example, in repetitive bending test, the entire device was encapsulated in mylar bags often used to manufacture pouch type secondary batteries. The electrode on PI was bonded to cured PDMS by using a thin layer of uncured PDMS as glue. The electrode was then cut in half to comprise the bottom and top electrodes of the energy-harvesting device. The two electrodes were placed on top of each other, separated by a layer of electrolyte-soaked separator (Celgard 2500) and were sealed using uncured PDMS on the sides.

To eliminate any possible difference in electrochemical potential or composition between the top and bottom electrode, the two electrodes were left connected in short-circuit via external wire for at least 2 h.

Bending tests were performed either by finger tapping or by servo motor (HS-7966HB, Hitec). A custom repeatable bending station was constructed using the servo motor, a 32 pitch gear (Servocity), a pinion and Arduino controller. The strain rate was ∼1% per second.

## Additional information

**How to cite this article:** Kim, S. *et al*. Electrochemically driven mechanical energy harvesting. *Nat. Commun.* 7:10146 doi: 10.1038/ncomms10146 (2016).

## Supplementary Material

Supplementary Information Supplementary Figures 1-7, Supplementary Notes 1-6 and Supplementary References

## Figures and Tables

**Table 1:** The predicted and measured voltage according to the radius of curvature values.

Radius of curvature (mm)	Hydrostatic stress (GPa)	*V*_predicted_ (mV)	*V*_measured_ (mV)
13.4	0.010	1.9	1.8
9.4	0.015	2.8	2.5
5.3	0.026	4.9	5.1
4.2	0.033	6.2	5.9
2.7	0.051	9.6	8.9
1.7	0.081	15.2	22.5

**Table 2:** Comparison of our energy harvester to piezoelectric generators in similar form factor.

	Active material thickness (μm)	Active area (cm^2^)	*V*_oc_ (V)	*I*_sc_ (μA)	Peak power (μW cm^−2^)	Energy output at 0.3 Hz (μJ cm^−2^ s^−1^)
Our device	0.25 × 2	0.63	0.023	14.5	0.53	0.476
PMN-PT	8.4	2.89	8.2	145	411	12.3
BaTiO_3_	250	12	3.2	0.35	0.093	0.00278
ZnO nanowire^15^	0.05	1	20	6	120	3.60
KNLN	250	9	12	1.2	1.60	0.0480
