# Supplementary material for: Electrochemically driven mechanical energy harvesting
Source: Nat Commun. 2016 Jan 6;7:10146. doi: 10.1038/ncomms10146 (PMC4729818; doi:10.1038/ncomms10146)
Supplement: Supplementary Information — Supplementary Figures 1-7, Supplementary Notes 1-6 and Supplementary References [file ncomms10146-s1.pdf]

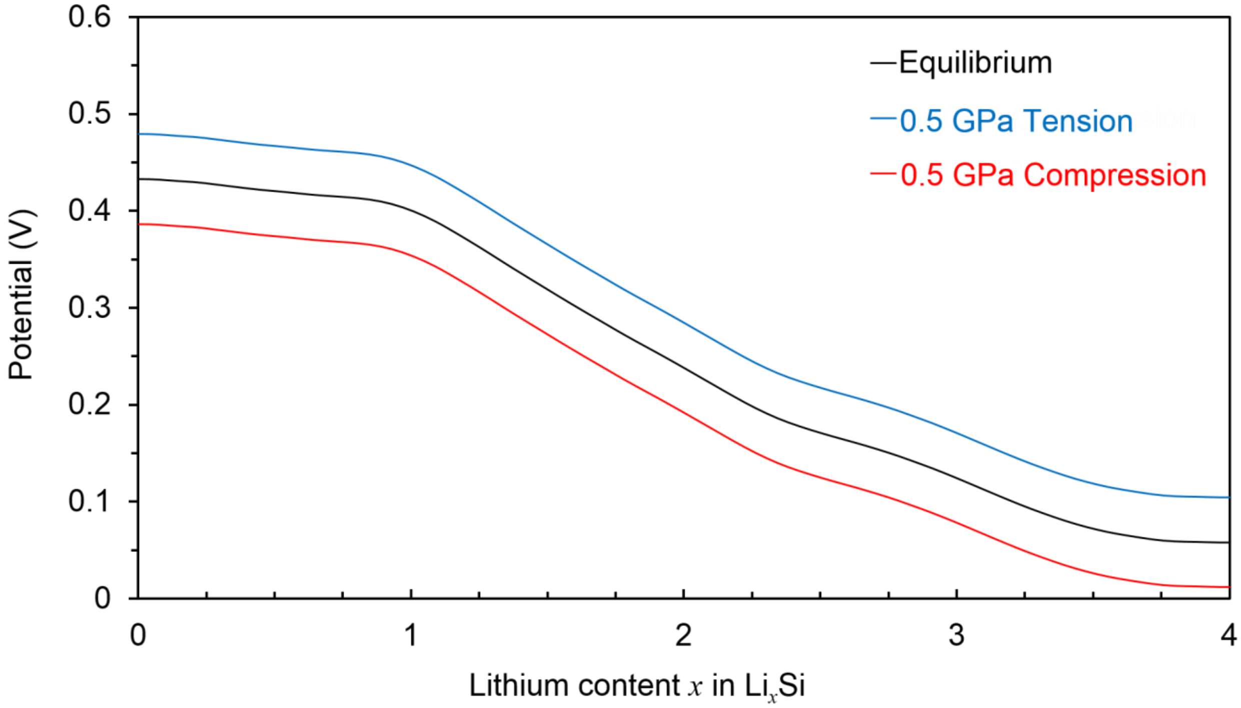

Supplementary Fig. 1. Electrochemical potential according to lithium content in Li_xSi alloy, with and without the effect of hydrostatic stress of -0.5GPa and +0.5GPa.

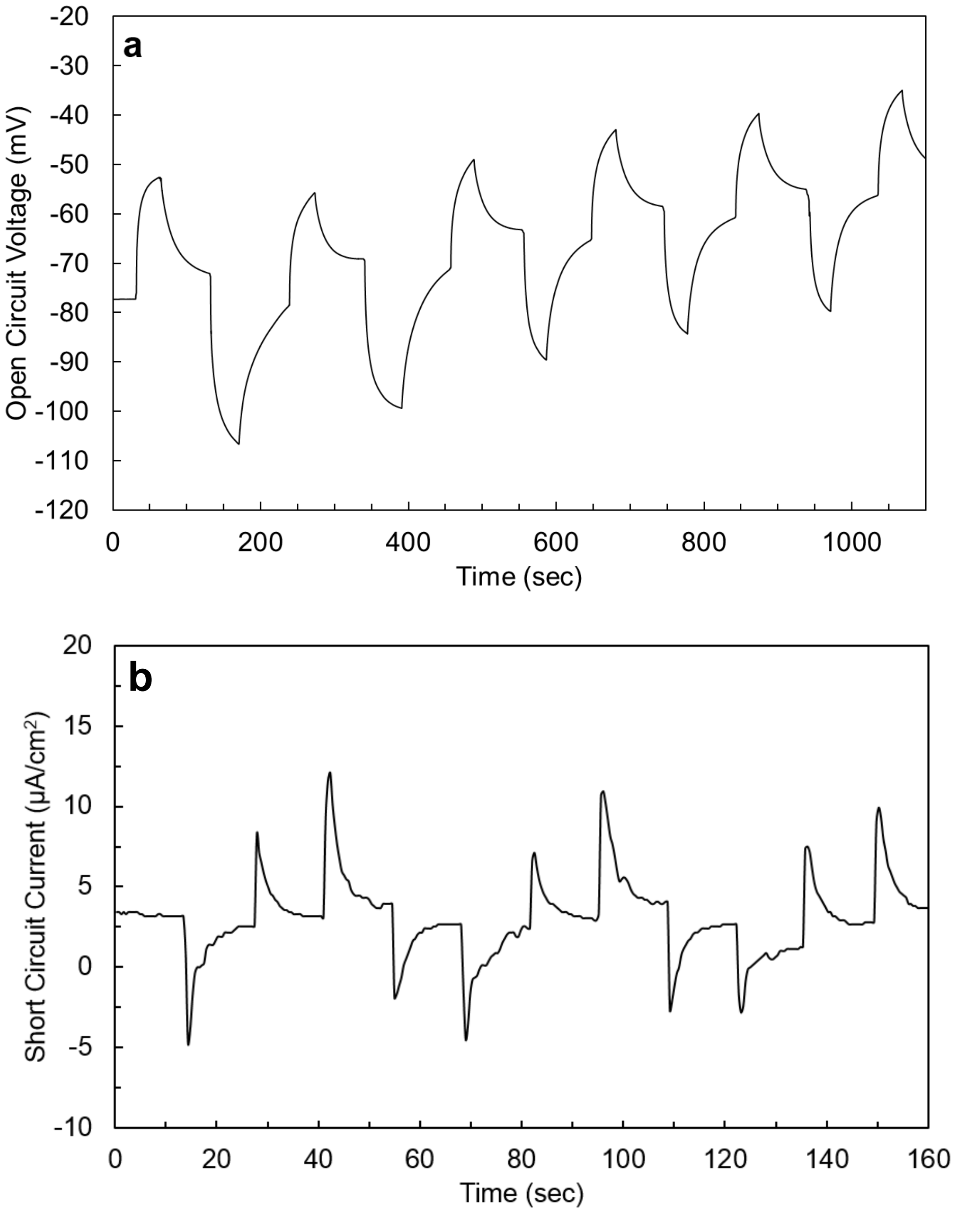

Supplementary Fig. 2. Output during bending the device in opposite directions at 1.7mm radius of curvature (a) Open circuit voltage data with voltage peak in alternating directions (b) Short circuit current during forward bending and backward bending while alternating bending directions.

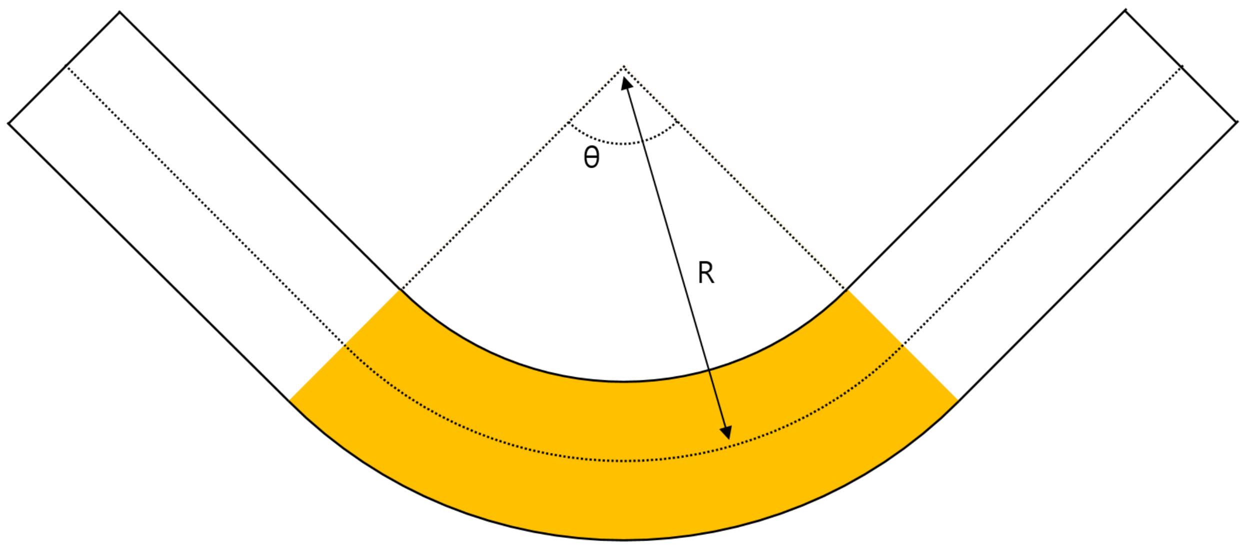

Supplementary Fig. 3. The bending geometry and estimation of the active area during bending

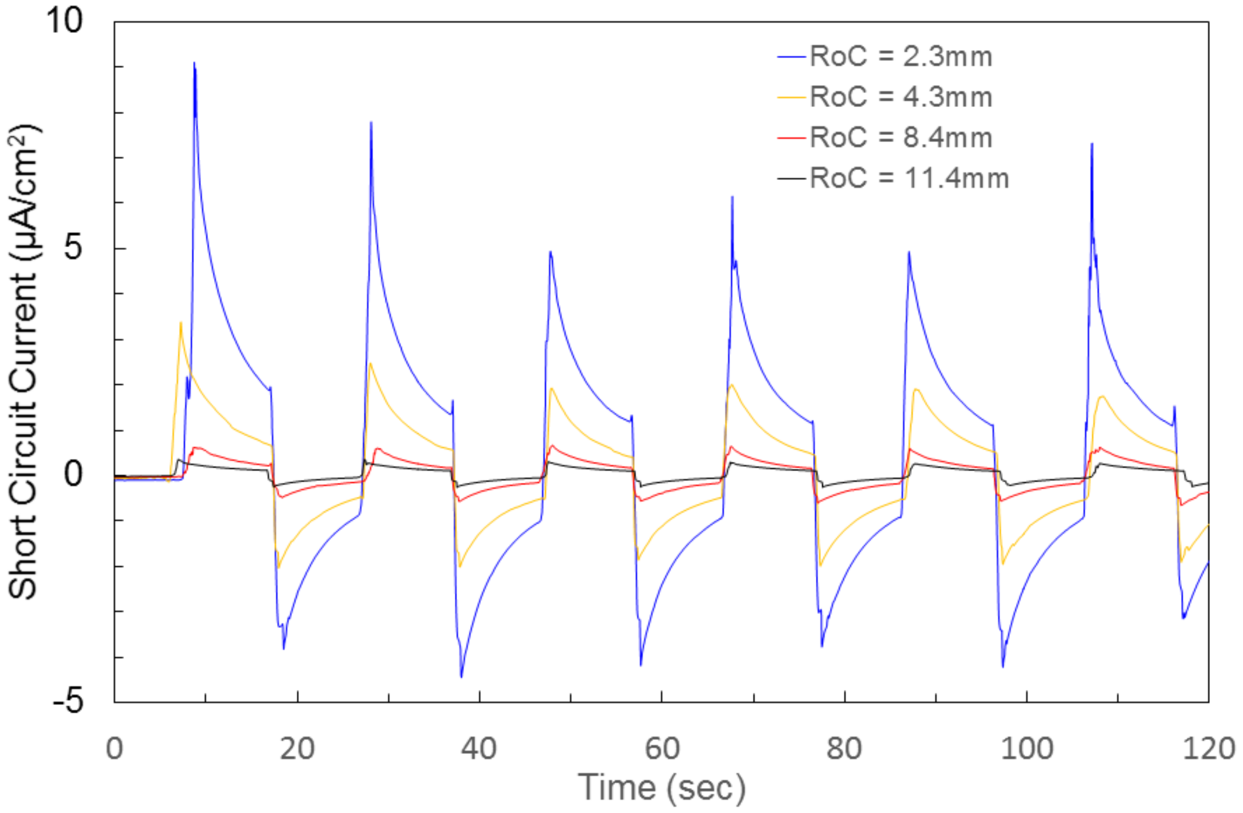

Supplementary Fig. 4. Short circuit current density measured at different radii of curvature.

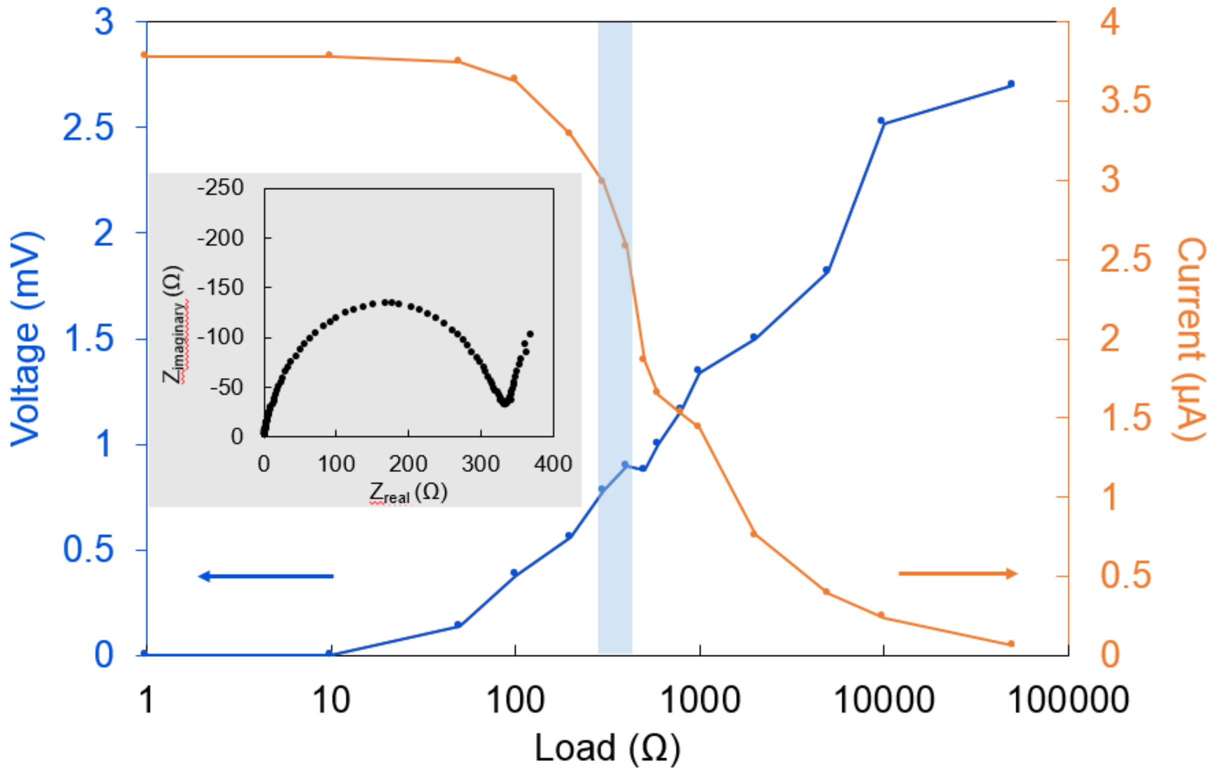

Supplementary Fig. 5. Voltage and current across external load varied from 1 to 50,000Ω. The external load that provides maximum power output is approximately 300Ω and is colored in light blue. The nested figure inside shows that the internal resistance of the device roughly matches the load that provides maximum power output.

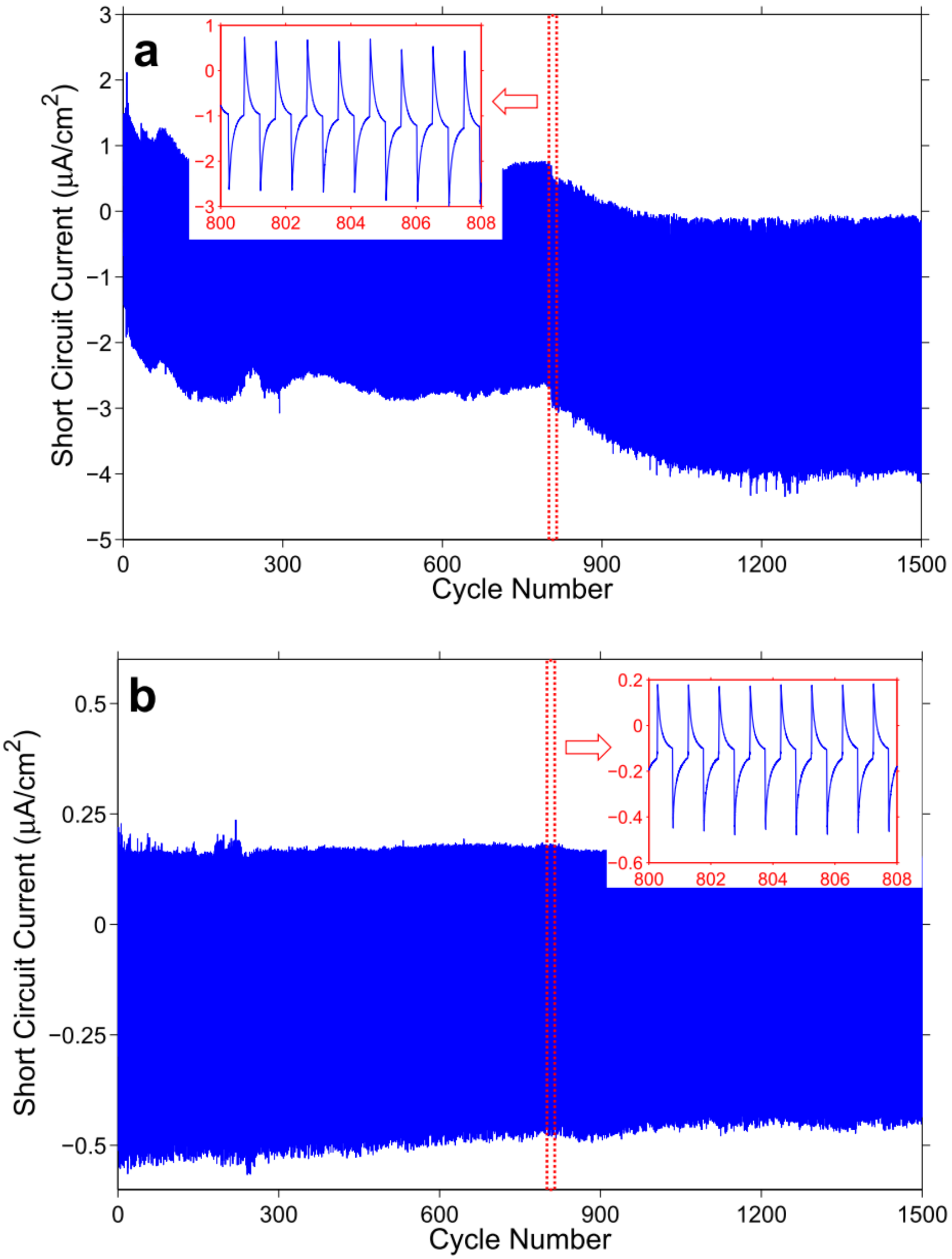

Supplementary Fig. 6. Short circuit current density during 1500 bending and unbending cycles measured at (a) 3.6mm radius of curvature and (b) at 11.4mm radius of curvature. The nested figure inside shows the zoomed-in view of the cycles between 800th and 807th bending.

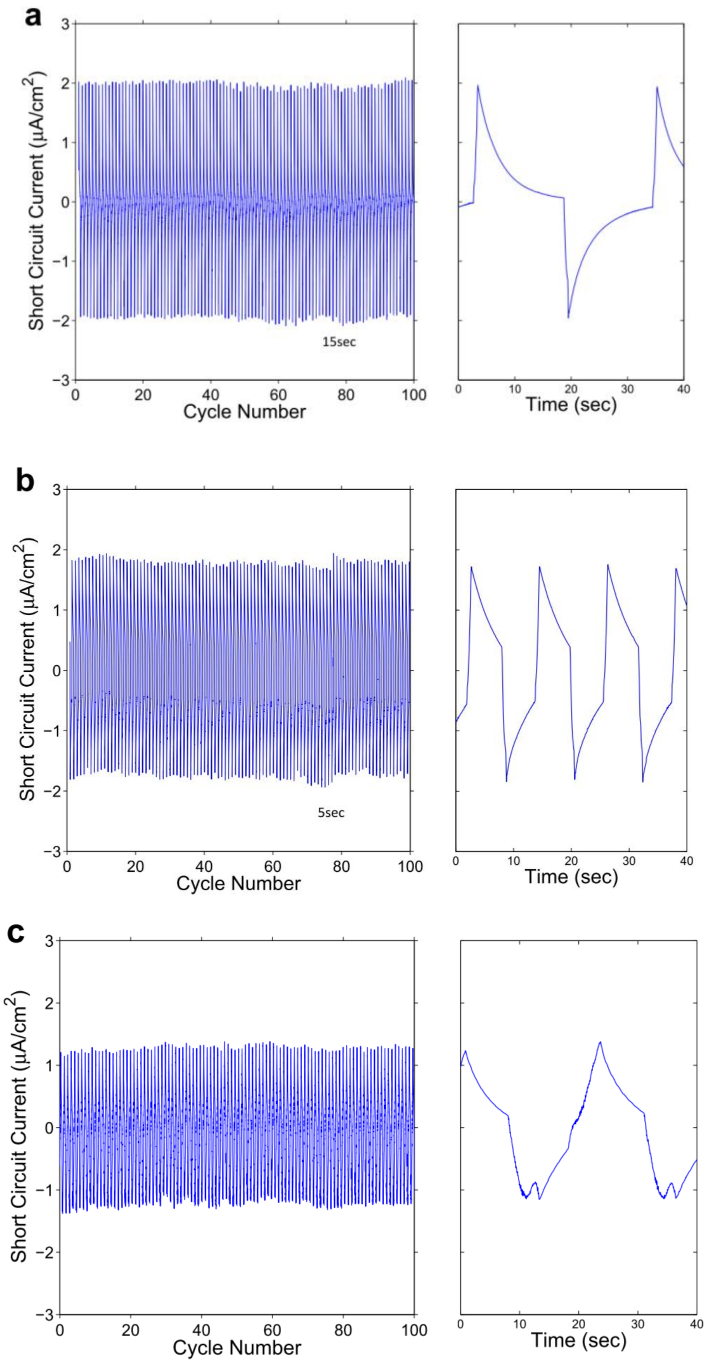

Supplementary Fig. 7. Short circuit current density measured at 3.6mm radius of curvature, at strain rate of 0.35% s^{-1} and bending frequency of (a) 0.07Hz, (b) 0.2Hz and (c) short circuit current density at strain rate of 0.07% s^{-1} and bending frequency of 0.2Hz. Figures on the left show 100 cycles at each bending frequency and figures on the right the zoomed-in view of the 50th cycle in normalized time.

Supplementary Notes

Supplementary Note 1. Stress contribution to the thermodynamic potential

The open circuit voltage between tensed and compressed Li_xSi electrodes can be expressed as the following with contributions from stress:

$$\Delta\phi = -\frac{RT}{F}\ln\left(\frac{\gamma_{Li}\chi}{1+\chi}\right) - \frac{\sigma_{jj}\Omega_{Li}}{F} \qquad (1)$$

where σ_{jj} stands for stress components, γ_{Li} the activity coefficient of Li, χ the normalized composition in Li_xSi, and Ω_{Li} the atomic volume of Li in the electrode material. Similar relations have been reported in previous reports.[1] The potential-composition relation without the contribution from stress for amorphous Li_xSi has been obtained by Density Functional Theory computations in a previous report.[2] Supplementary Fig. 1 illustrates the potential-composition behavior under no stress, 0.5GPa hydrostatic tension, and 0.5GPa hydrostatic compression. The data for non-stressed potential-composition relation was obtained from Vincent Chevrier.

Supplementary Note 2. Voltage and current data during alternating bending direction

Supplementary Fig. 2 illustrates the voltage and current data obtained during bending test in alternative direction. Bending is defined in two different directions, ‘forward’ direction and ‘backward’ direction. While bending in the forward direction, positive voltage peak is obtained. The peak voltage reaches up to 23mV at the radius of curvature of 1.7mm, in good agreement with the predicted value. Followed by the voltage decay, we observe a voltage peak in the opposite direction during backward bending. The bending experiment generates voltage peaks in a repeatable manner. The general increase in the background voltage may indicate that one electrode is accumulating more damage than the other as the unit is being bent in alternating direction in a severe manner. For instance, the initially compressed electrode may result in more

plastic flow compared to the other during the experiment, since the initial damage was more significant. The current signal follows similar manner to the one directional bending experiment, however, now exhibits the forward and backward current for alternating bending direction. The first peak in negative peak amplitude is followed by the positive peak from backward current. The third peak in positive peak is the result of bending the unit in the opposite direction, followed by its backward current. Similar to the one directional bending experiment, the full width at half maximum reaches up to 4.3 seconds.

Supplementary Note 3. The bending geometry and the amount of lithium inside the affected area

The amount of electrode material affected during the bending experiment can be computed based on the geometry with respect to the radius of curvature (R) and bending angle (θ). The bending geometry in this study and affected area are illustrated in Supplementary Fig. 3. The amount of silicon (N_{Si}, number of moles) is computed with respect to the thickness of the electrode (t), width of the device (w), density of silicon (ρ), and molar mass of silicon (m_{Si}).

$$N_{Si} = \frac{(t \times R\theta \times w) \times \rho}{m_{Si}} \quad (2)$$

Since the composition in Li_xSi alloy is precisely known from the lithiation profile, we may obtain N_{Li} by simply multiplying the normalized alloy composition to N_{Si}. For our experiment performed in Figure 1, plugging in the following numbers results in $N_{Si} = 3.9 \times 10^{-7}$.

t = 75nm, w = 2cm, R = 0.2cm, θ = 90°

Since the thin film is lithiated to $Li_{3.1}Si$ composition, the number of moles of lithium in the affected area is $N_{Si} = 1.2 \times 10^{-6}$. The final thickness of the thin film is estimated assuming perpendicular volume expansion to the substrate direction, as observed in previous reports.[3] The

volume expansion is estimated based on results of previous reports, resulting in 249nm $Li_{3.1}Si$ thin film.[3] This gives the one-side volume of the affected area to be $1.56\times10^{-5}\,cm^3$. This number may vary depending on the radii of curvature. Supplementary Fig. 4 illustrate the short circuit current according to the bending radii of curvature.

Supplementary Note 4. Estimation of the experimental efficiency

The experimental energy harvesting efficiency of our device can be estimated as the following:

$$\eta \equiv \frac{U_{\text{electrical}}}{U_{\text{strain}}} = \frac{\int J^2 R \cdot dt}{\int_0^L \frac{EI(\omega'')^2 dx}{2}} \tag{3}$$

where J stands for current, R resistor, E the Young's modulus, and I the second moment of inertia. The maximum electrical energy output is achieved at an external load comparable to the internal resistance. Supplementary Fig. 5 illustrates the voltage and current across external loads with varying load from 1Ω to 50,000Ω. The radius of curvature during bending was 9mm. The load that provides maximum power output is approximately 300Ω and is colored in light blue. This is significantly lower than many other types of mechanical energy harvesters, on the order of 10-100MΩ.[4,5] The nested figure demonstrates the Nyquist plot of the device and shows that the internal resistance of the device approximately matches with the load that provides the maximum power output.

The strain energy stored during bending the device can be estimated as the following.

$$U_{\text{strain}} = \sum_{component} \int_0^L \frac{EI(\omega'')^2 dx}{2} = \sum_{component} \frac{\theta EI}{2R} \tag{4}$$

Assuming a three layer (Li_xSi electrode – polymer separator – Li_xSi electrode) assembly, we obtain the estimated experimental efficiency of 0.62%. The Young's modulus of electrolyte soaked separator is directly measured and reported to be 0.35GPa in the literature.[6]

The loss in efficiency may be understood from 1) dissipative modes in U_{strain} and 2) resistive and kinetic loss in $U_{\text{electrical}}$. We have shown in the main text that only a fraction of the strain energy input is converted to electrical energy, due to the presence of the deviatoric stress tensor and also self-discharge between flat and non-flat regions. Contribution from vibration and frictional loss is also expected to reduce the efficiency despite a small amount.

Resistive and kinetic energy loss from $U_{\text{electrical}}$ is also expected. The presence of internal resistance brings resistive energy loss to the internal resistance. Also, as pointed out in the main text, the amount of actual charge transferred per bending is estimated to be approximately 10% of the generating capacity. This may be due to various reasons including self-discharge to a non-bent region, SEI layer formation at the electrode surface, charge transfer resistance at the electrode surface, or kinetic limitations from slow Li^+ ion diffusion. In most cases, the causes of these losses may be engineered and optimized through various techniques such as porous electrode design or artificial SEI layer design. We believe there is an ample room for further development and improvement.

Supplementary Note 5. Short circuit current density during 1500 bending and unbending cycles.

Repeated bending tests of 1500 cycles were performed at three different radii of curvature (3.6mm, 4.0mm and 7.4mm). Supplementary Fig. 6 illustrates the data. During the tests, we find that the background current (average current value between forward peak and backward peak) moves towards zero, as expected from chemical potential differences. This is true for 7.4mm and 4.0mm radii of curvature, but not true when cycled at 3.6mm radius of curvature. During cycling at 3.6mm radius of curvature, the background current fluctuates and eventually decreases to -

2μA from the original value of zero. The background current is expected to occur from inhomogeneous lithiation profile during sample preparation. The fluctuating and decreasing background current may indicate that plastic deformation accumulates on one electrode more severely than the other, causing the background current to fluctuate and eventually move out of equilibrium. With the current electrode design and packaging, we find from these durability tests that 3.6mm is the optimum radius of curvature, balancing between the power output and electrode stability

We also performed the durability test while alternating the bending direction between positive and negative bending. We observe that the overall current peaks are lower than that obtained from single bending direction but with occasional unexpectedly high peaks, indicating unstable mechanical response. The fluctuation in average current is also more severe than the single bending direction. We observe that such instability eventually leads to reduced peak height after 1000 cycles despite at 4.0mm radius of curvature.

Supplementary Note 6. Effect of strain rate and frequency in bending

The effect of strain rate and bending frequency is studied while bending at 3.6mm radius of curvature. Supplementary Fig. 7 illustrates the result. In order for clear comparison, the background current was removed. When the strain rate is maintained at 0.35% s^{-1} and bending frequency is varied from 0.2Hz to 0.07Hz, we do not observe any noticeable change in short circuit current peak height or change in durability during 100 cycles. When the bending frequency is maintained at 0.2Hz and strain rate is varied from 0.35% s^{-1} to 0.07% s^{-1}, we observe that the short circuit current peak shape widens during bending and peak height

decreases. As mentioned in the main text, however, the total amount of charge transferred during each bending cycle remains almost identical.

Supplementary References

1. Sheldon, B. W., Soni, S. K., Xiao, X. & Qi, Y. Stress contributions to solution thermodynamics in Li-Si alloys. *Electrochemical and Solid-State Letters* **15,** A9–A11 (2011).
2. Chevrier, V. L. & Dahn, J. R. First Principles Model of Amorphous Silicon Lithiation. *Journal of The Electrochemical Society* **156,** A454 (2009).
3. Beaulieu, L. Y., Eberman, K. W., Turner, R. L., Krause, L. J. & Dahn, J. R. Colossal Reversible Volume Changes in Lithium Alloys. *Electrochemical and Solid-State Letters* **4,** A137 (2001).
4. Park, K.-I. *et al.* Highly-Efficient, Flexible Piezoelectric PZT Thin Film Nanogenerator on Plastic Substrates. *Advanced Materials* **26,** 2514–2520 (2014).
5. Fan, F.-R., Tian, Z.-Q. & Lin Wang, Z. Flexible triboelectric generator. *Nano Energy* **1,** 328–334 (2012).
6. Cannarella, J. *et al.* Mechanical Properties of a Battery Separator under Compression and Tension. *Journal of The Electrochemical Society* **161,** F3117–F3122 (2014).
